# The Dimeric Form of 1,3‐Diaminoisoquinoline Derivative Rescued the Mis‐splicing of *Atp2a1* and *Clcn1* Genes in Myotonic Dystrophy Type 1 Mouse Model

**DOI:** 10.1002/chem.202001572

**Published:** 2020-10-06

**Authors:** Jun Matsumoto, Masayuki Nakamori, Tatsumasa Okamoto, Asako Murata, Chikara Dohno, Kazuhiko Nakatani

**Affiliations:** ^1^ Department of Regulatory Bioorganic Chemistry The Institute of Scientific and Industrial Research Osaka University 8-1 Mihogaoka Ibaraki 567-0047 Japan; ^2^ Department of Neurology Graduate School of Medicine Osaka University 2-2 Yamadaoka Suita 565-0871 Japan

**Keywords:** CUG repeat, DM1, mis-splicing, rescue, small molecule

## Abstract

Expanded CUG repeat RNA in the dystrophia myotonia protein kinase (*DMPK*) gene causes myotonic dystrophy type 1 (DM1) and sequesters RNA processing proteins, such as the splicing factor muscleblind‐like 1 protein (MBNL1). Sequestration of splicing factors results in the mis‐splicing of some pre‐mRNAs. Small molecules that rescue the mis‐splicing in the DM1 cells have drawn attention as potential drugs to treat DM1. Herein we report a new molecule **JM642** consisted of two 1,3‐diaminoisoquinoline chromophores having an auxiliary aromatic unit at the C5 position. **JM642** alternates the splicing pattern of the pre‐mRNA of the *Ldb3* gene in the DM1 cell model and *Clcn1* and *Atp2a1* genes in the DM1 mouse model. *In vitro* binding analysis by surface plasmon resonance (SPR) assay to the r(CUG) repeat and disruption of ribonuclear foci in the DM1 cell model suggested the binding of **JM642** to the expanded r(CUG) repeat in vivo, eventually rescue the mis‐splicing.

## Graphical abstract

**A new molecule JM642** alternates the splicing pattern of *Atp2a1* pre‐mRNA in the mouse model of myotonic dystrophy type 1 (DM1) to include the exon 22. In vitro binding assay showed that **JM642** bound to the CUG repeat RNA and disrupt the RNA foci in the cell.

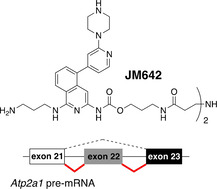

Molecules modulating the splicing pattern of genes in DM1 cells have drawn attention as potential drugs treating this devastating neurological disorder.[^1^, ^2^, ^3^, ^4^] DM1 is an autosomal dominant neuromuscular disorder, characterized by myotonia (delayed relaxation of muscles after contraction), progressive weakness, cardiac conduction defects, and cognitive impairments. The aberrant expansion of the CTG repeat in the 3′ untranslated regions of the *DMPK* gene is the cause of the disease.[^5^, ^6^, ^7^] The transcript of the *DMPK* gene with the long CUG repeat sequesters the RNA‐binding proteins, such as the splicing factor MBNL1 in the nucleus.[^8^, ^9^] As a consequence, several genes in DM1 cells showed different splicing patterns from those observed in the wild type cells.[^10^, ^11^] In the splicing of pre‐mRNA of the LIM domain binding 3 (*Ldb3*) gene, exon 11 is excluded from mRNA by about 80 % in the wild type, but exon 11‐included mRNA is produced about 50 % in DM1 cells.[^3^, ^12^] In the splicing of pre‐mRNAs encoding muscle‐specific chloride channel (*Clcn1*), the mRNA without exon 7a is dominant in wild type, whereas exon 7a‐included mRNA is abundant in DM1 cells.^[13]^ Similarly, splicing of pre‐mRNA of the *Atp2a1* gene coding sarcoplasmic reticulum calcium‐ATPase 1 (SERCA1) produces mRNA containing exon 22 in the wild type cells, whereas mRNA without exon 22 predominates in DM1 cells.^[14]^ Misregulated alternative splicing is a fundamental molecular feature of DM1, having good potential to function as biomarkers of severity and therapeutic response.^[11]^

These differences in the splicing patterns between the wild type and DM1 encouraged studies focused on the modulation of the splicing patterns. Besides prominent approaches using oligonucleotides,^[15]^ several groups have reported small molecules binding to the CUG repeats and modulating the splicing pattern in DM1 cells.[^16^, ^17^, ^18^, ^19^, ^20^, ^21^, ^22^, ^23^, ^24^, ^25^, ^26^, ^27^, ^28^, ^29^] We here report that the dimeric form of 1,3‐diaminoisoquinoline derivative **JM642** (Figure 1) rescued the mis‐splicing in *Ldb3* pre‐mRNA in the DM1 cell model and *Clcn1* and *Atp2a1* pre‐mRNAs in DM1 mouse model in a dose‐dependent manner. SPR assay showed the binding of **JM642** to the r(CUG)_9_‐immobilized sensor surface, and **JM642** led to the disruption of ribonuclear foci in DM1 cell model expressing r(CUG)_800_ repeat, demonstrating that **JM642** would be a useful molecular tool for the deeper understanding of the pathogenesis of DM1 and studies on the therapeutic potential of small molecules targeting DM1.

**Figure 1:**
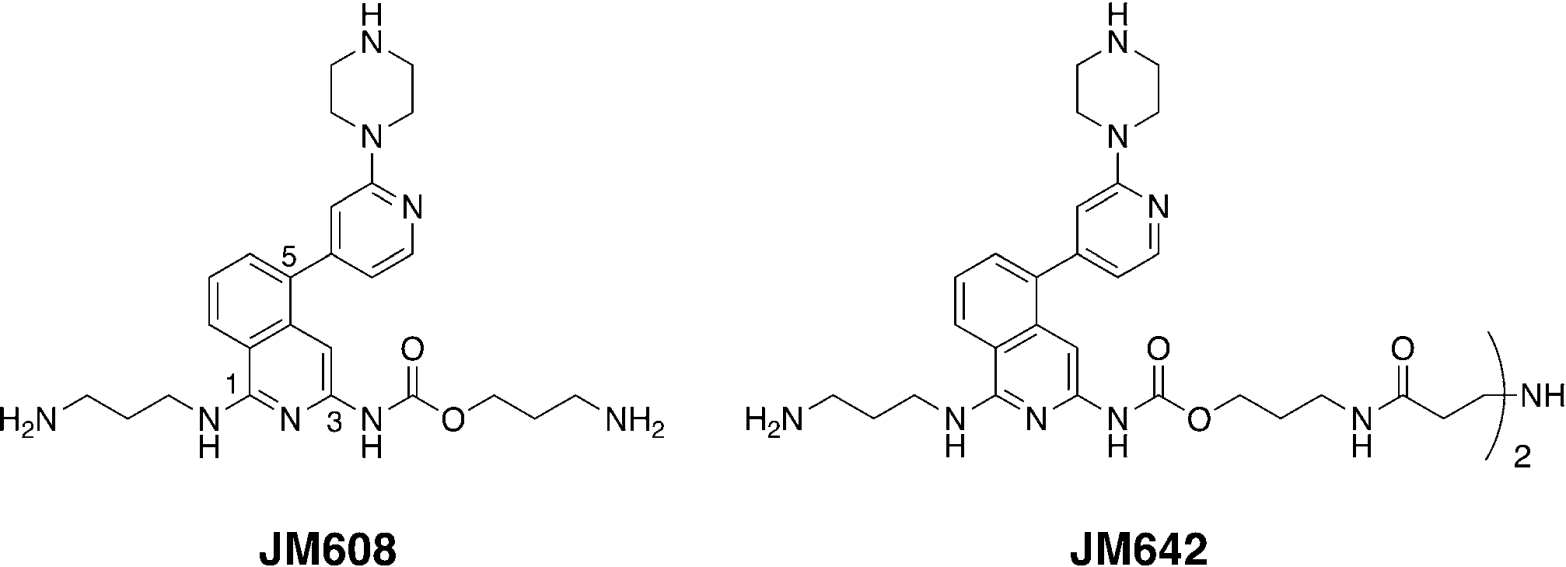
Chemical structures of **JM608** and the dimeric form **JM642**.

We have reported different types of molecules that bind to r(CUG) repeat and modulate the alternative splicing in DM1 cells.[^30^, ^31^, ^32^, ^33^] After structure–activity studies on small molecules targeting the r(CUG) repeat, we revisited 1,3‐diaminoisoquinoline derivatives with an additional aromatic unit at the C5 position and found a monomeric 1,3‐diaminoisoquinoline ligand **JM608** and its dimeric form **JM642**. (Figure 1) While the detail of structure–activity studies will be reported elsewhere, in brief, the substituent at the C5 position of the 1,3‐diaminoisoquinoline chromophore showed a significant effect on the binding to the CUG repeat RNA. **JM608** was synthesized by Suzuki–Miyaura cross‐coupling^[34]^ of the 1‐amino‐5‐bromo‐3‐chloroisoquinoline derivative **3** with a piperazine‐substituted pyridinyl pinacol boronic ester **8** followed by Buchwald‐Hartwig cross‐coupling^[35]^ of the resulting **4** at the C3 position with the Boc‐protected carbamate **9**. (Scheme 1) Deprotection of all Boc groups in **5** furnished the synthesis of **JM608. JM642** was obtained by coupling of **4** with a Cbz‐protected carbamate **10**, deprotection of the Cbz group in **6**, dimerization of **7** with a pentafluorophenyl‐activated biscarboxylic acid **11**,^[36]^ and deprotection of the Boc groups.

**Scheme 1:**
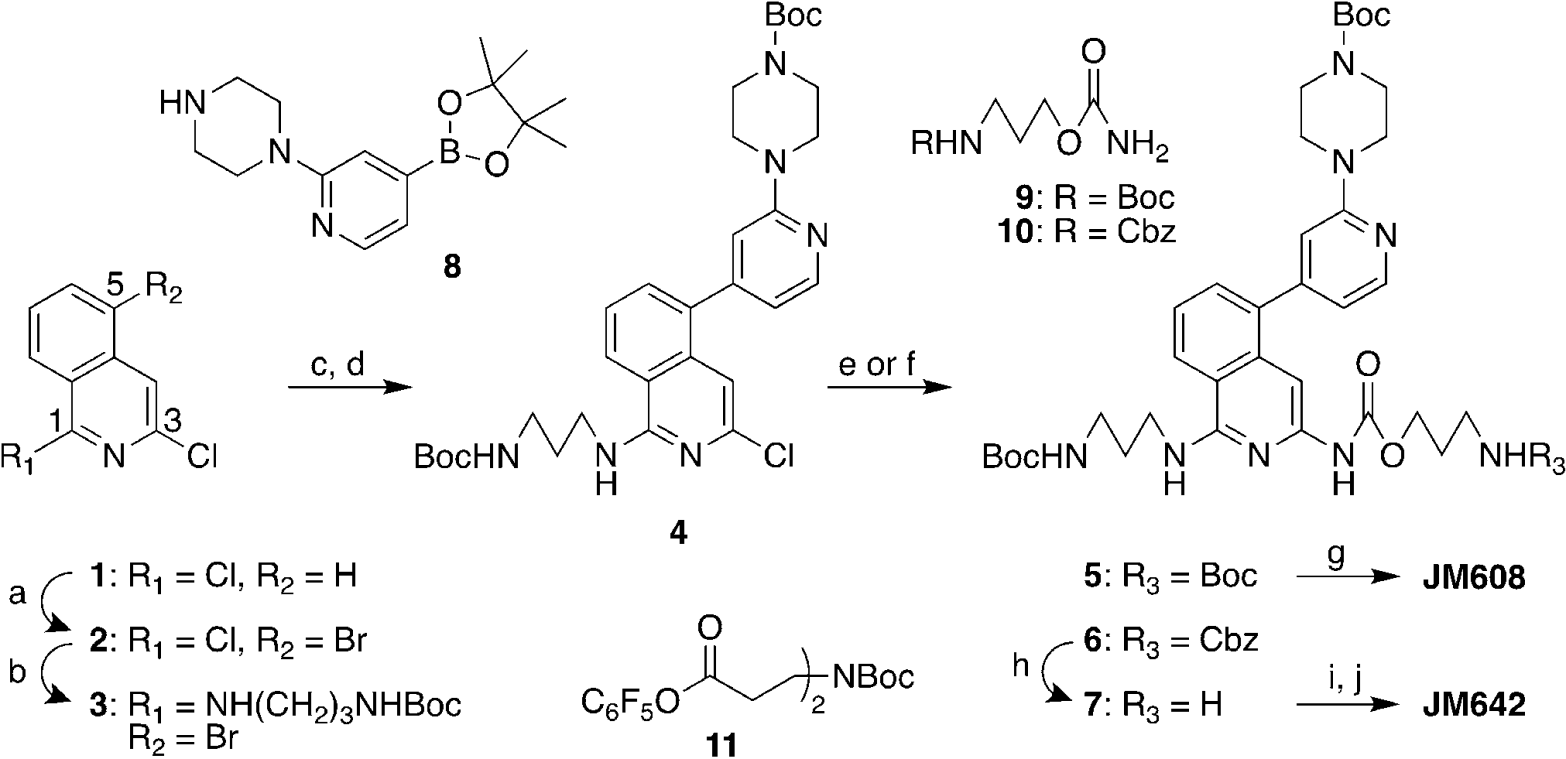
Synthetic scheme of **JM608** and **JM642**. (a) NBS, H_2_SO_4_, MeCN, r.t., 3 days, 44 %. (b) *N*‐Boc‐propanediamine, diisopropylethylamine, 1,4‐dioxane, reflux, overnight, 54 %. (c) **8**, Pd(PPh_3_)_4_, K_2_CO_3_, 1,4‐dioxane, H_2_O, Ar, 80 °C, 14 h. (d) Boc_2_O, r.t., 1 h, 86 % for two steps. (e) **9**, XPhos Pd G3, Cs_2_CO_3_, 1,4‐dioxane, Ar, reflux, 15 h, 39 %. (f) **10**, XPhos Pd G3, Cs_2_CO_3_, 1,4‐dioxane, Ar, reflux, 15 h, 52 %. (g) 4 m HCl in AcOEt, CHCl_3_, r.t., 1 h, 90 %. (h) H_2_, Pd/C (10 wt %), MeOH, r.t., 1 day, 74 %. (i) **11**, triethylamine, CHCl_3_, 50 °C, 1 day, 89 %. (j) 4 m HCl in AcOEt, CHCl_3_, r.t., 1 h, 90 %.

The effect of **JM608** and **JM642** on alternative splicing was investigated on pre‐mRNA of the *Ldb3* gene in the C2C12 DM1 cell model conditionally expressing r(CUG)_800_ repeat RNA.^[37]^ (Figure 2 a) In the control cells without expression of r(CUG)_800_, the percentage of exon 11 exclusion in the *Ldb3* gene was about 81±1.7 %, while the fraction in the DM1 cell model expressing r(CUG)_800_ was 53±1.9 % (Figure 2 b). After the treatment of the DM1 cell model with **JM642** for two days, the mis‐splicing of *Ldb3* pre‐mRNA was significantly rescued in a dose‐dependent manner, increasing exon 11 exclusion up to 77±2.5 % with 80 μM. The observed rescue effect of **JM642** on the mis‐splicing is statistically significant (**P<0.01) at the concentrations higher than 30 μM. The effect of a monomer **JM608** on the recovery in mis‐splicing was 58±1.9 % at 80 μM. For the reference, cytotoxicity of **JM608** and **JM642** to the C2C12 DM1 cell model was not apparent over the treatment range (data not shown).

**Figure 2:**
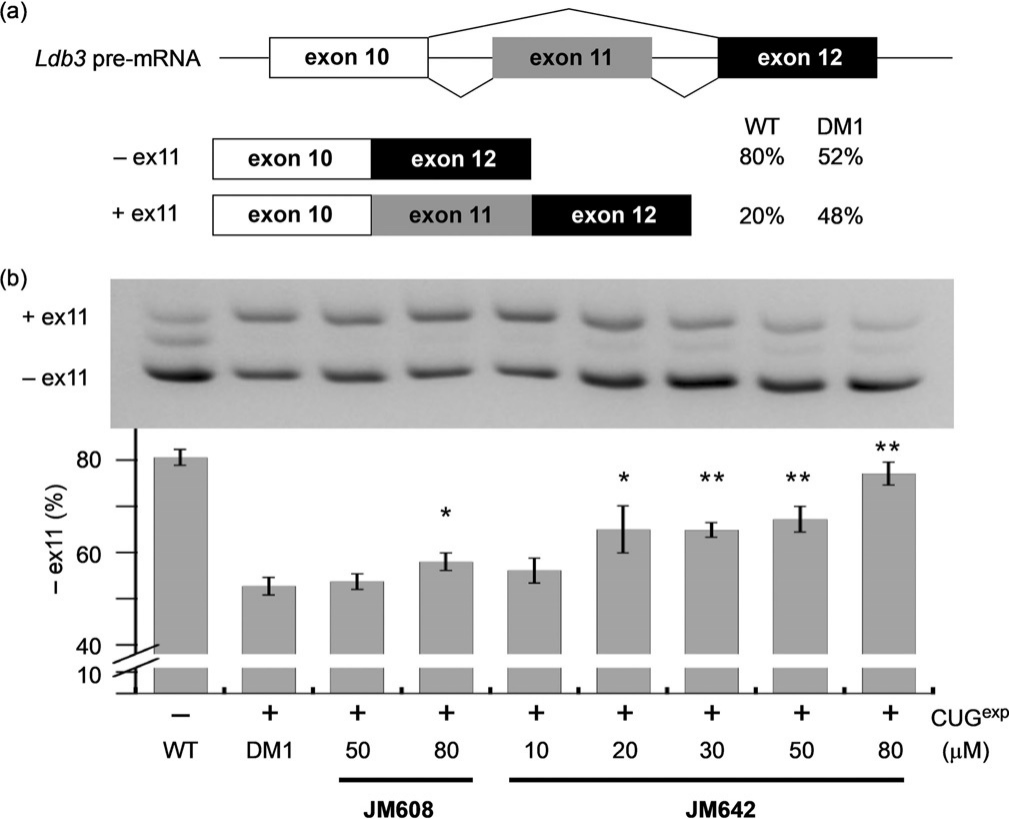
**JM608** and **JM642** rescued the mis‐splicing in the C2C12 DM1 cell model. (a) Schematic representation of alternative splicing of *Ldb3* pre‐mRNA in WT and DM1 cells. (b) Gel image (upper panel) of RT‐PCR products for inclusion and exclusion of *Ldb3* exon 11 and bar graph (lower panel) representing the percentage of exon 11 exclusion. The r(CUG)_800_ expressing cells were treated with different concentrations of **JM608** and **JM642**. *P<0.05 and **P<0.01. Error bars indicated SDM.

We then investigated the effect of **JM642** on the mis‐splicing of pre‐mRNAs in the DM1 mouse model (*HSA* ^LR^), which expresses r(CUG)_220_ and exhibits the mis‐splicing of *Clcn1* and *Atp2a1* pre‐mRNAs.^[38]^ **JM642** (10 mg kg^−1^ or 20 mg kg^−1^ per day) was administrated to the *HSA* ^LR^ mice (*n=*3 in each group) by daily intraperitoneal (i.p.) injection for five days. The fraction of exon 7a exclusion for the *Clcn1 gene* was 85±0.53 % for the wild type mice and 44±2.4 % for the *HSA* ^LR^ mice. (Figure 3 a). Mis‐splicing of *Clcn1* has been suggested to cause myotonia.^[13]^ Treatment of the *HSA* ^LR^ mice with i.p. **JM642** (10 and 20 mg kg^−1^) rescued the mis‐splicing in the *Clcn1* gene, leading to an exclusion rate of 61±2.3 % (*P*=0.03) and 70±2.3 % (*P*=0.01), respectively, although an improvement of phenotypic myotonia was not apparent due to the partial rescue of splicing. The rescue effect of **JM642** was also observed in *Atp2a1* mis‐splicing. In the wild‐type mice, the inclusion rate for exon 22 is 100±0 %, whereas the inclusion fraction of exon 22 in the *HSA* ^LR^ mice was 16±2.4 % (Figure 3 b). After administration of 10 and 20 mg kg^−1^ of **JM642**, the inclusion rate improved to 32±2.5 % (*P*<0.05) and 74±6.0 % (*P*<0.01), respectively. Toxicity was not observed within the mouse model over this treatment range. These results demonstrated the rescue effect of **JM642** on mis‐splicing of *Clcn1* and *Atp2a1* pre‐mRNAs in DM1 in vivo.

**Figure 3:**
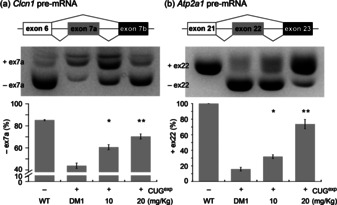
**JM642** rescued splicing defects in (a) *Clcn1* and (b) *Atp2a1* pre‐mRNAs in DM1 mouse model (HSA^LR^). Representative gel images of RT‐PCR products for *Clcn1* exon 7a (top) upon treatment of **JM642** and the corresponding bar graphs (bottom). The (CUG)_220_ expressing mice were treated with the indicated concentration of **JM642** by daily i.p. injection for five days. *N*=3 for experimental, control, and wild type. *P<0.05 and **P<0.01. Error bars indicated SEM.

Having observed the significant effect of **JM642** and somewhat moderate effect of **JM608** on the rescue in mis‐splicing of genes in DM1 cell and mouse models, we have investigated the origin of these biological effects. The current hypothesis on the expected therapeutic effects of small molecules in the treatment of DM1 stems from the competitive binding of small molecules with RNA‐binding proteins to the aberrantly expanded CUG repeat RNA in the nucleus.[^1^, ^2^, ^3^] To know if **JM608** and **JM642** could fit this hypothesis, we looked at the binding of these molecules to the CUG repeat RNA with the SPR assay. The biotin‐labeled r(CUG)_9_ repeat RNA and r(CCG)_9_ repeat RNA as control were immobilized through the tri‐ethylene glycol linker to the avidin‐coated sensor surface, and the analyte molecule was sequentially added with the increased concentration to the surface (single cycle kinetic analysis).

The SPR response curves obtained for **JM608** and **JM642** from the same sensor surface of r(CUG) repeat RNA were quite different in terms of the shape of the curve, which characterizes the association and dissociation kinetics as well as the affinity. The SPR profiles obtained for **JM608** showed the rectangular shape indicating a rapid association and dissociation kinetics. (Figure 4 a) The lowest concentration necessary for the significant SPR response under the conditions was 63 nm. The apparent dissociation constant (*K*_d(app)_) of **JM608** to the r(CUG)_9_ repeat RNA was determined 1.2 μm based on the assumed 1:1 binding isotherm.

**Figure 4:**
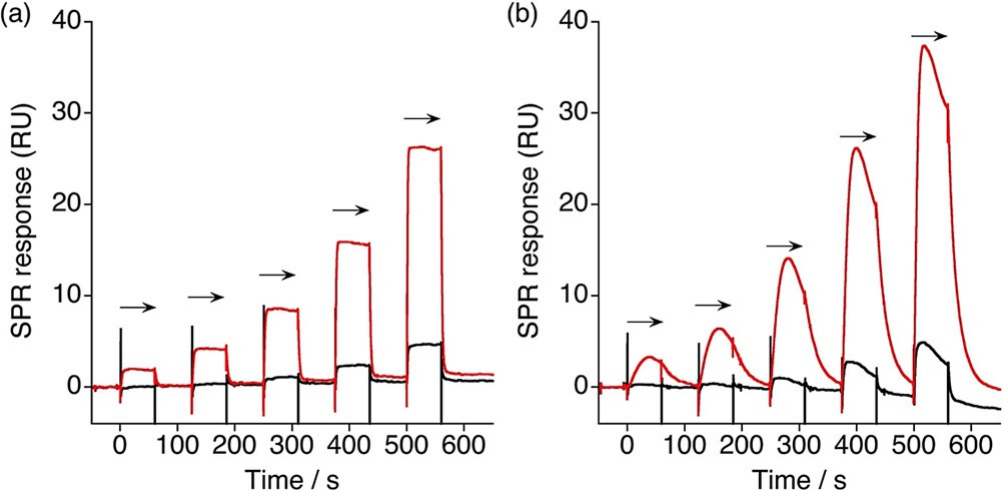
SPR single cycle kinetic analyses of ligand binding to the r(CUG)_9_ (red) and r(CCG)_9_ (black). **JM608** and **JM642** were applied to the RNA‐immobilized surface for 60 seconds (shown with solid arrows), and the sensor surface was subsequently washed by the running buffer for 60 s before the next injection of the ligand. (a) **JM608** was added stepwise at concentrations of 0.063, 0.13, 0.25, 0.5, and 1.0 μm. (b) **JM642** was added stepwise at concentrations of 6.3, 12.5, 25.0, 50.0, and 100 nm.

In contrast, SPR response curves obtained for **JM642** were quite characteristic, showing a broad parabola shape without any plateau region. (Figure 4 b) The lowest concentration of **JM642** for producing a significant SPR response was 6.3 nm, which is one order of magnitude smaller than that of **JM608**, suggesting a positive effect of dimerization of **JM608** on the binding to the CUG repeat. The parabola shape observed for the response curves is unique and is likely due to the dimeric form. The SPR response increased as the duration in applying **JM642** prolonged. However, the SPR signal started to decrease while **JM642** was kept applying to the surface. In general, SPR responses reach the plateau or steadily increase due to the equilibrium shift toward the ligand‐bound state from the free unbound state in the bulk solution. The characteristic phenomena in SPR analysis of **JM642** are likely due to conformational changes on the **JM642**‐CUG RNA complex on the surface after initial complex formation. The significant effects of the linker length and structure connecting two isoquinoline chromophores on the binding to r(CUG)_9_ observed in the SPR analysis may support the above speculation. (Figure S1 in the Supporting Information) SPR responses on the r(CCG) repeat RNA surface were weak for both **JM608** and **JM642**, even at 1.0 and 0.1 μm, respectively.

To gain insight into the possibility of competitive binding of **JM642** with RNA‐binding proteins on CUG repeat RNA, we have investigated the disruption of the ribonuclear foci in DM1 patient‐derived myoblast cells by **JM642** treatment. Untreated DM1 myoblasts showed the formation of ribonuclear foci (Figure 5 a). The percentage of cells showing foci positive nucleus was 41±7.0 % among 255 cells examined. Upon treatment with 30 μm **JM642**, the number of cells showing the foci positive nucleus dropped to 6.7±1.3 % among 286 cells counted. Since the FISH probes capture the CUG repeat RNA, the CUG repeat RNA was suggested to dissociate from the aggregates forming foci in the cell nucleus upon **JM642** treatment.

**Figure 5:**
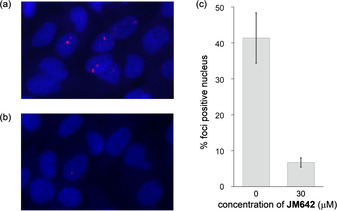
**JM642** disrupted ribonuclear foci in DM1 myoblasts. FISH showing foci in CUG^exp^ RNA (red) in nuclei (blue) of DM1 myoblast with (a) non‐treated and (b) treatment of **JM642** (30 μm) for two days. (c) Histogram showing the percentage of cells with nuclear foci of CUG^exp^. Mean ± SD, *n*=3 or more. The number of cells counted was 255 for no treatment and 286 for 30 μm **JM642** treatment.

In summary, a newly developed small molecule **JM642**, a dimeric form of 5‐substituted‐1,3‐diaminoisoquinoline derivative **JM608**, rescued the mis‐splicing in both DM1 cell and mouse models. The increased affinity and the different modes of the binding of **JM642**, as compared to **JM608** in SPR assay in vitro likely attributed to the difference of biological activity in the DM1 cell model. Disruption of the ribonuclear foci in the DM1 cell model also supported the possibility of **JM642**‐binding competitively with RNA‐binding proteins. Overall, **JM642** could be a useful molecular tool for the studies on the biological responses induced by expanded CUG repeat.

## Experimental Section

### Studies on rescue effect of small molecules on the DM1 cell model

A conditional cell model for the analysis of MBNL1 splicing regulatory activity has been established, as reported previously.^[37]^ Briefly, C2C12 mouse muscle cells were co‐transfected with pLC16 containing 800 CTG repeats and plasmid PhiC31° encoding PhiC31 integrase (Addgene, Cambridge, MA). Transfection was performed using Nucleofector technology (Lonza, Basel, Switzerland) according to the manufacturer's program B‐32. Stably transfected clones were selected with puromycin (1.25 μg mL^−1^). Transcription across the expanded repeat was activated by Cre recombinase‐mediated excision of a transcription terminator cassette. C2C12 cells with recombination were selected using hygromycin B (300 μL mL^−1^). RNA was harvested after 2 days of incubation with **JM608** and **JM642**. RNA extraction and analysis of the splicing pattern were carried out as described below. WST‐1 assay was performed according to the manufacturer's instructions (Roche, Basel, Switzerland).

### Studies on rescue effect of small molecules on the DM1 mouse model

Mouse handling and experimental procedures were performed following the Osaka University guidelines for the welfare of animals and were approved by the institutional review board. Homozygous *HSA* ^LR^ transgenic mice of line 20b (FVB inbred background) were described previously.^[38]^ Gender‐ and age‐matched (<3 months old) mice were treated with **JM642** at indicated dose and period by daily i.p. injection. After treatments, mice were sacrificed, and the rectus femoris (quadriceps) muscle was obtained for splicing analysis. RNA extraction and analysis of the splicing pattern were carried out as described below.

### RNA extraction and splicing analysis

Total RNA extraction from model cells, cDNA synthesis, and polymerase chain reaction (PCR) amplification were performed as described previously.^[39]^ The PCR products were separated by agarose gel electrophoresis, and the gel was stained with GelRed (Biotium, Hayward, CA). The gel was imaged using a Typhoon laser fluorimager (GE Healthcare, Pittsburgh, PA) and the products quantified using ImageQuant (GE Healthcare).

## Conflict of interest

The authors declare no conflict of interest.

## Supporting information

As a service to our authors and readers, this journal provides supporting information supplied by the authors. Such materials are peer reviewed and may be re‐organized for online delivery, but are not copy‐edited or typeset. Technical support issues arising from supporting information (other than missing files) should be addressed to the authors.

Supplementary
