# Supplementary material for: The Dimeric Form of 1,3‐Diaminoisoquinoline Derivative Rescued the Mis‐splicing of Atp2a1 and Clcn1 Genes in Myotonic Dystrophy Type 1 Mouse Model
Source: Chemistry. 2020 Oct 6;26(63):14305–9. doi: 10.1002/chem.202001572 (PMC7702137; doi:10.1002/chem.202001572)
Supplement: Supplementary file 1 — Supplementary [file CHEM-26-14305-s001.pdf]

Chemistry–A European Journal

Supporting Information

The Dimeric Form of 1,3-Diaminoisoquinoline Derivative Rescued the Mis-splicing of *Atp2a1* and *Clcn1* Genes in Myotonic Dystrophy Type 1 Mouse Model

Jun Matsumoto,[a] Masayuki Nakamori,[b] Tatsumasa Okamoto,[a] Asako Murata,[a] Chikara Dohno,[a] and Kazuhiko Nakatani*[a]

Contents

1. Synthesis of JM608 and JM642
2. Surface plasmon resonance (SPR) binding assay
3. Cell viability assays
4. Fluorescence in situ hybridization
5. Structure-Binding studies on JM642 derivatives with different linker length

Synthesis of JM608 and JM642

General

Reagents and solvents were purchased from standard suppliers and used without further purification. Reactions were monitored with TLC plates precoated with Merch Silica Gel 60 F254. Spots were visualized with UV light or ninhydrin. Wako gel C-200 was used for silica gel flash chromatography. High performance liquid chromatography (HPLC) was performed by a Gilson 811C Dynamic Mixer system with a UV detector set at 254 nm using a Cosmosil $5C_{18}$-MS-II column (150 x 20 mm) with a dual solvent system of 0.1% $AcOH/H_2O$ (solvent A) and MeCN (solvent B). ^1H NMR and ^{13}C NMR spectra were measured with ECS400 (JEOL), ECA600 (JEOL) and Avance III 700 (BRUKER). The chemical shifts are expressed in ppm relative to a residual solvent as an internal standard. ESI mass spectra were recorded on a JEOL AccuTOF-T100N mass spectrometer.

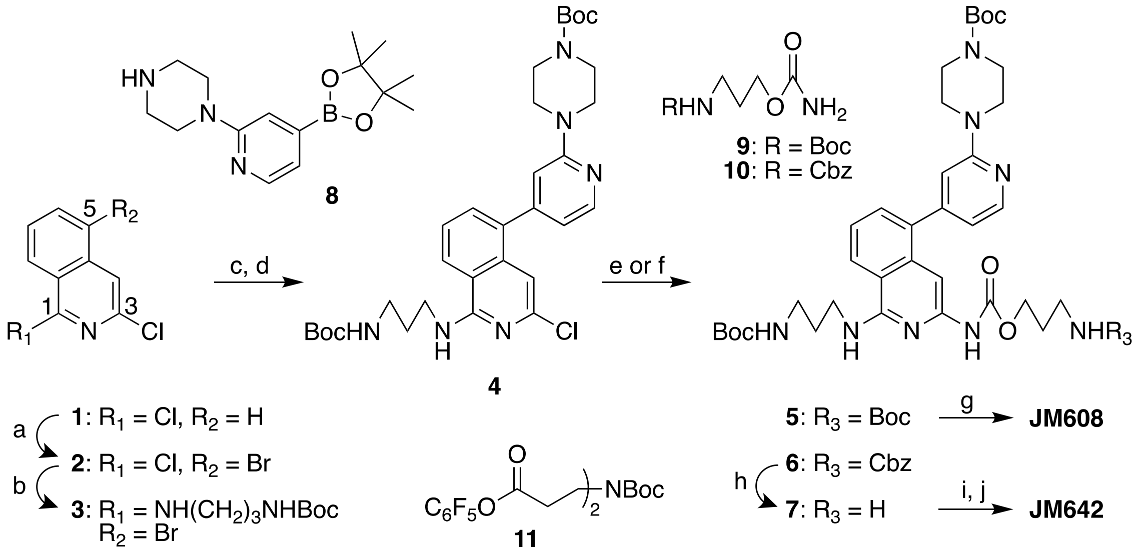

Scheme 1. Synthetic scheme of **JM608** and **JM642**. (a) NBS, H_2SO_4, MeCN, r.t., 3 days, 44%, (b) *N*-Boc-propanediamine, diisopropylethylamine, 1,4-dioxane, reflux, overnight, 54% (c) **8**, $Pd(PPh_3)_4$, K_2CO_3, 1,4-dioxane, H_2O, Ar, 80 ºC, 14 h, (d) Boc_2O, r.t., 1 h, 86% for two steps (e) **9**, XPhos Pd G3, Cs_2CO_3, 1,4-dioxane, Ar, reflux, 15 h, 39% (f) **10**, XPhos Pd G3, Cs_2CO_3, 1,4-dioxane, Ar, reflux, 15 h, 52% (g) 4 N HCl in AcOEt, $CHCl_3$, r.t., 1 h, 90% (h) H_2, Pd/C (10wt%), MeOH, r.t., 1 day, 74%

(i) **11**, triethylamine, $CHCl_3$, 50 °C, 1 day, 89%. (j) 4 M HCl in AcOEt, $CHCl_3$, r.t., 1 h, 90%.

5-Bromo-1,3-dichloroisoquinoline (2)

1,3-Dichloroisoquinoline (**1**) (2.0 g, 10.1 mmol) and *N*-bromosuccinimide (2.3 g, 12.9 mmol) were mixed in dry acetonitrile (50 mL), then sulfuric acid (2 mL) was added dropwise and the mixture was stirred at room temperature for 3 days. The produced solid was separated by filtration and washed with hexane. The white solid was dried to give **2** (1.2 g, 44%). ^1H NMR (600 MHz, $CDCl_3$): δ = 8.30 (d, *J* = 8.2 Hz, 1 H), 8.03 (d, *J* = 7.6 Hz, 2 H), 7.53 (m, 1 H). ^{13}C NMR (150 MHz, $CDCl_3$): δ = 151.6, 144.9, 138.4, 136.0, 129.0, 126.9, 126.5, 121.1, 119.5. HRMS (ESI) m/z: calcd. for $[C_9H_4{}^{79}Br^{35}Cl_2N + Na]^+$, 297.8796; found 297.8798.

***tert*-Butyl (3-((5-bromo-3-chloroisoquinolin-1-yl)amino)propyl)carbamate 3**

2 (1.0 g, 3.6 mmol) was dissolved in 1,4-dioxane (8 mL), then diisopropylamine (1 mL) and *N*-Boc-1,3-propanediamine (3 mL) were added and the mixture was refluxed overnight. The resulting solution was neutralized with the aqueous ammonium chloride solution and extracted with dichloromethane. The organic phase was dried with anhydrous $MgSO_4$ and concentrated under the reduced pressure. The residue was purified by column chromatography on silica gel eluted with 1% MeOH/$CHCl_3$ to give **3** (1.5 g, 54%) as white solid. ^1H NMR (600 MHz, $CDCl_3$): *δ* = 7.87 (d, J = 8.2 Hz, 1 H), 7.83 (d, J = 7.6 Hz, 1 H), 7.27 (t, J = 8.2 Hz, 1 H), 7.21 (s, 1 H), 6.79 (s, 1 H), 5.05 (t, J = 5.8 Hz, 1 H), 3.70 (q, J = 6.0 Hz, 2 H), 3.25 (q, J = 5.5 Hz, 2 H), 1.79 (quin, J = 5.8 Hz, 2 H), 1.48 (s, 9 H). ^{13}C NMR (150 MHz, $CDCl_3$): δ = 157.3, 155.9, 146.5, 138.1, 134.4, 126.0, 121.9, 121.1, 118.1, 106.9, 79.8, 37.7, 37.2, 29.9, 28.6. HRMS (ESI) m/z: calcd. for $[C_{17}H_{29}{}^{79}Br^{35}ClN_3O_2 + Na]^+$, 436.0398; found 436.0398.

***tert*-Butyl 4-(4-(1-((3-((*tert*-butoxycarbonyl)amino)propyl)amino)-3-chloroisoquinolin-5-yl)pyridin-2-yl)piperazine-1-carboxylate (4)**

A mixture of **3** (400 mg, 0.96 mmol), 1-(4-(4,4,5,5-tetramethyl-1,3,2-dioxaborolan-2-yl)pyridin-2-yl)piperazine (**8**) (335 mg, 1.16 mmol), potassium carbonate (400 mg, 2.89 mmol), and $Pd(PPh_3)_4$ (111 mg, 96 μmol) in a solvent mixture of 1,4-dioxane (12 mL) and water (1 mL) under an atmosphere of argon was stirred at 80 °C for 14 h. The reaction mixture was cooled to room temperature, then Boc_2O (1.5 mL) was added and stirred for 1 h. the reaction mixture was neutralized with the aqueous NH_4Cl solution, and extracted with CH_2Cl_2. The organic phase was dried with anhydrous $MgSO_4$, and concentrated under the reduced pressure. The residue was purified by column chromatography on silica gel eluted with 30% AcOEt/Hexane to give **4** (493 mg, 86%) as pale yellow solid. ^1H NMR (600 MHz, $CDCl_3$): δ = 8.27 (d, J = 2.4 Hz, 1 1 H), 7.93 (t, J = 4.7 Hz, 1 H), 7.48 (d, J = 4.8 Hz, 2 H), 6.85 (s, 1 H), 6.71 (q, J = 2.1 Hz, 1 H), 6.68 (t, J = 5.8 Hz, 1 H), 6.65 (s, 1 H), 5.14 (t, J = 6.2 Hz, 1 H), 3.72 (q, J = 6.0 Hz, 2 H), 3.58 (t, J = 4.8 Hz, 8 H), 3.26 (q, J = 6.0 Hz, 2 H), 1.79 (q, J = 5.5 Hz, 2 H), 1.48 (s, 18H). ^{13}C NMR (150 MHz, $CDCl_3$): δ = 159.6, 157.2, 155.9, 155.0, 149.3, 148.1, 145.5, 137.3, 136.7, 130.7, 125.3, 122.4, 117.0, 115.2, 108.1, 105.5, 80.1, 79.6, 45.2, 44.0, 42.9, 37.6, 37.2, 30.0, 28.6, 28.5. HRMS (ESI) m/z: calcd. for $[C_{30}H_{42}{}^{35}ClN_6O_4 + H]^+$, 597.2951; found 597.2955.

***tert*-Butyl 4-(4-(3-(((3-((*tert*-butoxycarbonyl)amino)propoxy)carbonyl)amino)-1-((3-((*tert*-butoxycarbonyl)amino)propyl)amino)isoquinolin-5-yl)pyridin-2-yl)piperazine-1-carboxylate 5**

A mixture of **4** (144 mg, 241 μmol), *tert*-butyl (3-(carbamoyloxy)propyl)carbamate (**9**) (158 mg. 722 μmol), cesium carbonate (235 mg, 722 μmol), and XPhos Pd G3 (20 mg, 24 μmol) in dry 1,4-dioxane (9 mL) under an atmosphere of argon was refluxed for 15 h. The reaction mixture was cooled to room temperature, diluted with ethyl acetate, filtered through a short plug of silica, and concentrated *in vacuo*. The residue was purified by column chromatography on silica gel eluted with 40% AcOEt/Hexane to give **5** (73.2 mg, 39%) as pale yellow solid. ^1H NMR (600 MHz, $CDCl_3$): δ = 8.28 (d, J = 5.6 Hz, 1 H), 7.84 (d, J = 8.2 Hz, 1 H), 7.50 (s, 1 H), 7.46 (d,

J = 7.3 Hz, 1 H), 7.34 (t, J = 7.7 Hz, 1 H), 7.12 (s, 1 H), 6.78 (d, J = 4.7 Hz, 2 H), 6.34 (s, 1 H), 5.10 (s, 1 H), 4.84 (s, 1 H), 4.16 (t, J = 6.0 Hz, 2 H), 3.65 (q, J = 6.2 Hz, 2 H), 3.58 (d, J = 26.2 Hz, 8 H), 3.26 (q, J = 5.7 Hz, 2 H), 3.18 (s, 2 H), 1.80 (m, 2 H), 1.78 (m, 2 H), 1.48 (s, 18 H), 1.42 (s, 9 H). ^{13}C NMR (150 MHz, $CDCl_3$): δ = 159.5, 157.0, 156.1, 155.2, 155.0, 153.1, 149.9, 148.0, 145.4, 137.4, 137.1, 130.7, 123.4, 122.2, 116.1, 115.2, 108.6, 92.6, 80.0, 79.6, 79.3, 62.7, 45.2, 44.1, 42.9, 37.6, 37.5, 37.3, 29.9, 29.5, 28.6, 28.6, 28.6. HRMS (ESI) m/z: calcd. for $[C_{40}H_{58}N_8O_8 + H]^+$, 779.4450; found 779.4443.

3-Aminopropyl (1-((3-aminopropyl)amino)-5-(2-(piperazin-1-yl)pyridin-4-yl)isoquinolin-3-yl)carbamate (JM608)

To a solution of **5** (8.5 mg, 10.8 μmol) in $CHCl_3$ (1 mL) was added ethyl acetate containing 4 M HCl (2 mL), and the reaction mixture was stirred at room temperature for 1 h. The solvent was evaporated to dryness to give **JM608** (5.7 mg, 90%) as yellow solid. The product was further purified by HPLC for the assay. ^{1}H NMR (600 MHz, D_2O): δ = 8.21 (d, J = 5.5 Hz, 1 H), 7.96 (d, J = 8.2 Hz, 1 H), 7.54 (d, J = 7.6 Hz, 1 H), 7.44 (t, J = 7.9 Hz, 1 H), 7.06 (s, 1 H), 6.94 (s, 1 H), 6.92 (d, J = 5.5 Hz, 1 H), 4.20 (t, J = 5.8 Hz, 2 H), 3.73 (t, J = 5.2 Hz, 4 H), 3.67 (t, J = 6.5 Hz, 2 H), 3.31 (t, J = 5.2 Hz, 4 H), 3.09 (t, J = 7.2 Hz, 2 H), 3.05 (t, J = 7.2 Hz, 2 H), 1.92 (m, 2 H), 1.91 (m, 2 H) ^{13}C NMR (150 MHz, D_2O): δ = 158.9, 156.1, 154.9, 150.8, 147.0, 144.5, 135.9, 135.9, 131.2, 124.2, 122.9, 116.6, 115.6, 109.9, 92.9, 62.7, 43.4, 43.1, 37.3, 36.9, 36.9, 27.1, 26.3. HRMS (ESI) m/z: calcd. for $[C_{25}H_{34}N_8O_2 + 2H]^{2+}$, 240.1475; found 240.1477.

***Tert*-butyl 4-(4-(3-(((3-(((benzyloxy)carbonyl)amino)propoxy)carbonyl)amino)-1-((3-((*tert*-butoxycarbonyl)amino)propyl)amino)isoquinolin-5-yl)pyridin-2-yl)piperazine-1-carboxylate 6**

A mixture of **4** (500 mg, 837 μmol), benzyl (3-(carbamoyloxy)propyl)carbamate (**10**) (317 mg. 1.26 mmol), cesium carbonate (818 mg, 2.51 mmol), and XPhos Pd G3 (71 mg,

84 μmol) in dry 1,4-dioxane (35 mL) under an atmosphere of argon was refluxed for 15 h. The reaction mixture was cooled to room temperature, diluted with ethyl acetate, filtered through a short plug of silica, and concentrated *in vacuo*. The residue was purified by column chromatography on silica gel eluted with 20-60% AcOEt/Hexane to give **6** (356 mg, 52%) as pale-yellow solid. ^{1}H NMR (600 MHz, $CDCl_3$): δ = 8.28 (d, J = 5.5 Hz, 1 H), 7.84 (d, J = 7.6 Hz, 1 H), 7.51 (s, 1 H), 7.47 (d, J = 6.9 Hz, 1 H), 7.32-7.36 (5 H), 7.30 (t, J = 4.1 Hz, 1 H), 7.10 (s, 1 H), 6.78 (d, J = 4.1 Hz, 2 H), 6.33 (s, 1 H), 5.18 (s, 1 H), 5.08 (s, 2 H), 4.18 (t, J = 5.8 Hz, 2 H), 3.65 (q, J = 5.5 Hz, 2 H), 3.61 (d, J =5.6 Hz, 4 H), 3.56 (d, J = 5.5 Hz, 4 H), 3.26 (d, J = 5.5 Hz, 4 H), 1.84 (t, J = 6.2 Hz, 2 H), 1.78 (t, J = 5.5 Hz, 2 H), 1.48 (s, 9 H), 1.48 (s, 9 H). ^{13}C NMR (150 MHz, $CDCl_3$): δ = 159.51, 156.97, 156.59, 155.22, 155.05, 153.14, 149.81, 148.06, 145.40, 137.40, 137.10, 136.69, 130.75, 128.61, 128.22, 128.19, 123.45, 122.15, 116.09, 115.13, 108.61, 92.67, 79.99, 79.67, 66.75, 62.65, 45.31, 43.98, 42.89, 37.95, 37.66, 37.37, 29.91, 29.49, 28.57. HRMS (ESI) m/z: calcd. for $[C_{43}H_{56}N_8O_8 + H]^+$, 813.4294; found 813.4298.

***tert*-Butyl 4-(4-(3-(((3-aminopropoxy)carbonyl)amino)-1-((3-((*tert*-butoxycarbonyl) amino)propyl)amino)isoquinolin-5-yl)pyridin-2-yl)piperazine-1-carboxylate 7**

6 (316 mg, 389 μmol) was dissolved in MeOH (170 mL), then Pd/C (10 wt%) (60 mg) was added. The mixture was stirred under hydrogen at room temperature for 1 day. The Pd/C was filtered through a short pad celite. After the solvent was concentrated, the residue was purified by column chromatography on amino-coated silica gel eluted with 2% MeOH/$CHCl_3$ to give **7** (219 mg, 74%) as pale-yellow solid. ^{1}H NMR (600 MHz, $CDCl_3$): δ = 8.28 (d, J = 4.8 Hz, 1 H), 7.83 (d, J = 8.2 Hz, 1 H), 7.52 (s, 1 H), 7.47 (d, J = 7.6 Hz, 1 H), 7.35 (t, J = 7.9 Hz, 1 H), 7.04 (s, 1 H), 6.79 (d, J = 5.5 Hz, 2 H), 6.28 (s, 1 H), 5.10 (s, 1 H), 4.21 (t, J = 6.2 Hz, 2 H), 3.66 (q, J = 6.2 Hz, 2 H), 3.61 (d, J = 5.9 Hz 4 H), 3.57 (d, J = 5.9 Hz, 4 H), 3.26 (q, J = 5.7 Hz, 2 H), 2.78 (t, J = 6.2 Hz, 2 H), 1.80 (m, 2 H), 1.79 (m, 2 H), 1.49 (s, 9

H), 1.48 (s, 9 H). ^{13}C-NMR (176 MHz, $CDCl_3$) δ 159.58, 156.95, 155.20, 155.03, 153.19, 149.85, 148.04, 145.46, 137.46, 137.13, 130.78, 123.43, 122.10, 116.04, 115.19, 108.57, 92.71, 79.99, 79.69, 63.03, 45.29, 44.11, 42.91, 38.96, 37.63, 37.32, 32.94, 29.95, 28.59. HRMS (ESI) m/z: calcd. for $[C_{35}H_{50}N_8O_6 + H]^+$, 679.3926; found 679.3930.

Di-*tert*-butyl 4,4'-((((10-(*tert*-butoxycarbonyl)-7,13-dioxo-2,18-dioxa-6,10,14-triazanonadecanedioyl)bis(azanediyl))bis(1-((3-((*tert*-butoxycarbonyl)amino)propyl) amino)isoquinoline-3,5-diyl))bis(pyridine-4,2-diyl))bis(piperazine-1-carboxylate) Boc-JM642

7 (50 mg, 73.7 μmol) and bis(perfluorophenyl) 3,3'-((*tert*-butoxycarbonyl)azanediyl) dipropionate (**11**) (19 mg, 32.5 μmol) was dissolved in $CHCl_3$ (1 mL), then triethylamine (47 μL, 338 μmol) was added and the mixture was stirred at 50 °C for 1 day. The reaction mixture was cooled to room temperature, and neutralized with the aqueous ammonium chloride solution, and extracted with chloroform. The organic phase was dried with anhydrous $MgSO_4$, and concentrated under the reduced pressure. The residue was purified by column chromatography on basic silica gel eluted with 1% MeOH/$CHCl_3$ to give **Boc-JM642** (51.9 mg, 89%) as pale-yellow solid. ^{1}H NMR (600 MHz, $CDCl_3$): δ = 8.25 (d, J = 5.5 Hz, 2 H), 7.84 (d, *J* = 8.2 Hz, 2 H), 7.50 (s, 2H), 7.44 (d, J = 6.9 Hz, 2 H), 7.39 (s, 2 H), 7.31 (t, J = 7.6 Hz, 2 H), 6.77 (d, J = 5.5 Hz, 4 H), 6.40 (s, 2 H), 5.16 (t, J = 6.2 Hz, 2 H), 4.11 (d, J = 5.5 Hz, 4 H), 3.63 (m, 4 H), 3.61 (m, 8 H), 3.57 (m, 8 H), 3.49 (m, 4 H), 3.27 (d, J = 4.8 Hz, 4 H), 3.22 (d, J = 6.2 Hz, 4 H), 2.42 (s, 4 H), 1.80 (t, J = 6.2 Hz, 4 H), 1.74 (t, J = 5.5 Hz, 4 H), 1.46 (s, 18 H), 1.45 (s, 18 H), 1.40 (s, 9 H). ^{13}C NMR (150 MHz, $CDCl_3$): δ = 159.47, 156.97, 155.86, 155.21, 155.03, 153.16, 149.83, 147.96, 145.50, 137.24, 137.01, 130.69, 123.35, 122.21, 116.06, 115.13, 108.61, 92.57, 80.30, 80.00, 79.56, 62.86, 45.33, 45.14, 42.85, 37.71, 37.42, 36.46, 36.07, 29.81, 28.94, 28.53, 28.48. HRMS (ESI) m/z: calcd. for $[C_{81}H_{115}N_{17}O_{16} + 2H]^{2+}$, 791.9427; found 791.9434.

((3,3'-Azanediylbis(propanoyl))bis(azanediyl))bis(propane-3,1-diyl) bis((1-((3-aminopropyl)amino)-5-(2-(piperazin-1-yl)pyridin-4-yl)isoquinolin-3-yl)carbamate) JM642

To a solution of **Boc-JM642** (51.9 mg, 32.8 μmol) in chloroform (2 mL) was added ethyl acetate containing 4 M HCl (4 mL), and the reaction mixture was stirred at room temperature for 1 h. The solvent was evaporated to dryness to give **JM642** (31.9 mg, 90%) as yellow solid. The product was further purified by HPLC for the assay. ^{1}H NMR (600 MHz, D_2O): δ = 7.88 (d, J = 4.8 Hz, 2 H), 7.49 (d, J = 8.2 Hz, 2 H), 7.09 (d, J = 6.9 Hz, 2 H), 6.97 (t, J = 7.6 Hz, 2 H), 6.74 (s, 2 H), 6.52 (d, J = 4.8 Hz, 2 H), 6.37 (s, 2 H), 3.88 (t, J = 5.8 Hz, 4 H), 3.50 (t, J = 5.8 Hz, 4 H), 3.40 (t, J = 4.8 Hz, 8 H), 3.16 (t, J = 4.9 Hz, 8 H), 3.10 (m, 4 H), 3.06 (t, J = 5.1 Hz, 4 H), 2.84 (t, J = 5.1 Hz, 4 H), 2.80 (t, J = 6.9 Hz, 4 H), 2.38 (t, J = 5.5 Hz, 4 H), 1.86 (m, 4 H), 1.62 (s, 4 H). ^{13}C NMR (176 MHz, D_2O): δ = 172.86, 158.28, 155.60, 154.72, 150.29, 146.64, 144.38, 135.39, 134.98, 131.10, 123.63, 122.62, 116.32, 115.24, 109.67, 91.98, 62.88, 43.51, 43.03, 42.97, 37.04, 36.67, 35.94, 32.01, 27.96, 27.17. HRMS (ESI) m/z: calcd. for $[C_{57}H_{76}N_{16}O_6 + 2H]^{2+}$, 541.8816; found 541.8821.

<u>Surface plasmon resonance (SPR) binding assay</u>

A streptavidin-coated sensor chip (SA chip, GE Healthcare) was washed with HBS-EP$^+$ buffer (10 mM HEPES pH 7.4, 0.15 M NaCl, 3 mM EDTA and 0.05% v/v Surfactant P20) for 6 min and then activated with three consecutive 1 min injection of 30 μL activation buffer (50 mM NaOH and 1 M NaCl). 5'-Biotinylated r(CUG)$_9$ and r(CCG)$_9$ (purchased from Thermo Fisher Scientific Inc.) was diluted to 0.1 μM with HEPES buffer (10 mM HEPES and 500 mM NaCl) and flowed onto the SA chip until immobilized response units (RU) reaching around 400 RU. The amount of r(CUG)$_9$ and r(CCG)$_9$ immobilized on the chip surface was 401 and 399 RU, respectively. Bindings of the ligands to the surface were analyzed by using a BIAcore T200 SPR system (GE Healthcare).

RNA used for the SPR studies.

	sequence	
$r(CUG)_9$	5′-biotin-TEG-CUGCUGCUGCUGCUGCUGCUGCUGCUG-3′	
$r(CCG)_9$	5′-biotin-TEG-CCGCCGCCGCCGCCGCCGCCGCCGCCG-3′	

Single-cycle kinetic analysis was carried out at 25 °C under the continuous flow of HBS-EP$^+$ buffer at a flow rate of 30 μL/min. The surface was conditioned by 120 sec exposure to analyse the association of ligands to the sensor chip. Each ligand was dissolved in HBS-EP$^+$ buffer at the concentration of 0.063, 0.125, 0.25, 0.50, and 1.0 μM (for $r(CUG)_9$), and the resulting solutions were sequentially injected over flow cells on the sensor surface for 60 sec at a flow rate of 30 μL/min in single-cycle mode. The obtained data was analysed using BIAcore T200 evaluation software, version 2.0, and kinetic parameters were determined by affinity method and curve fitting method of 1:1 binding mode.

Cell viability assays

Cell viability upon **JM608** and **JM642** treatments was assayed by the WST-8 reagent (DOJINDO laboratories, Japan) following the manufacturer’s instructions. HeLa cells were seeded into 96 well plate (at 5 x 10^3 cells per well) before incubating with ligands for 24 hours. Cell viability was determined after addition of ligands for 24 h. 1 h incubation after addition of 5 μL of WST-8, the absorbance at 450 nm was measured by EL808 Absorbance reader (BioTek$^{○,R}$). Cell viability was determined based on the absorbance relative to that for control well. Each condition was assayed in at least four replicate wells.

Fluorescence in situ hybridization

FISH for myoblast of DM1 patient was performed, as previously described. (M. Nakamori, G. Gourdon, C. A. Thornton, Mol. Ther. 2011, 19, 2222-2227) Fluorescence images were obtained by using a fluorescence microscope (Biozero BZ-9000 Keyence, Tokyo, Japan). The number of foci in the untreated cells was counted at least 70 nuclei in 3 independent experiments.

(a)

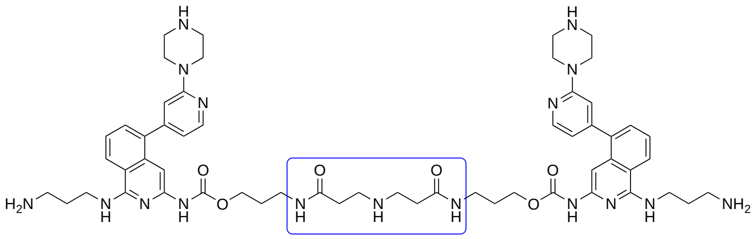

JM642

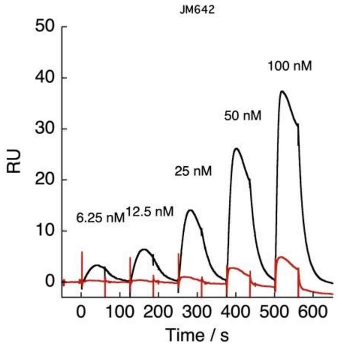

(b)

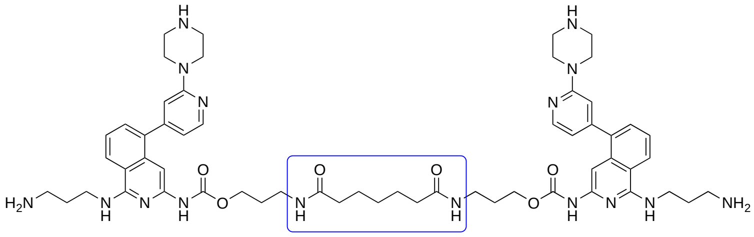

JM676

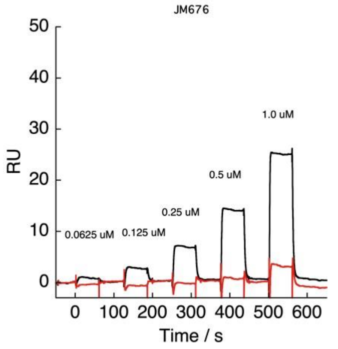

(c)

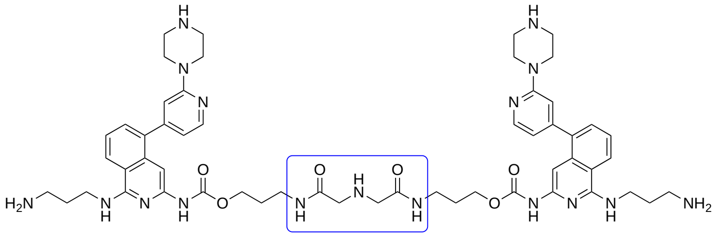

JM675

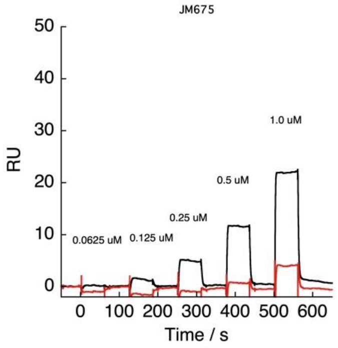

(d)

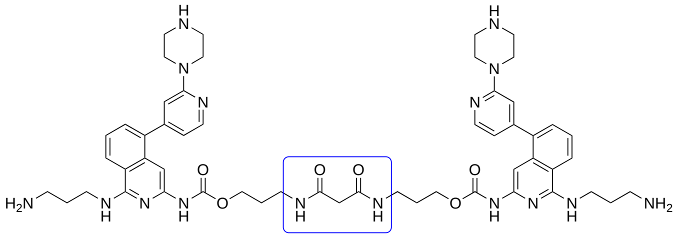

JM665

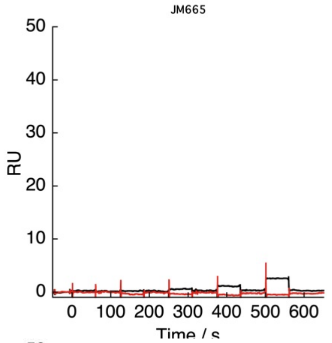

(e)

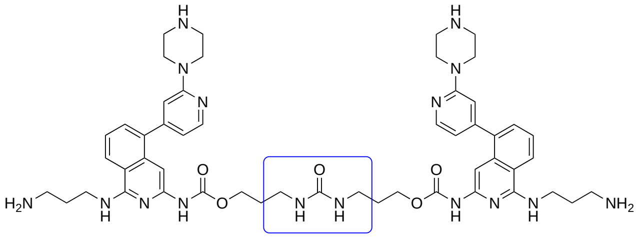

JM641

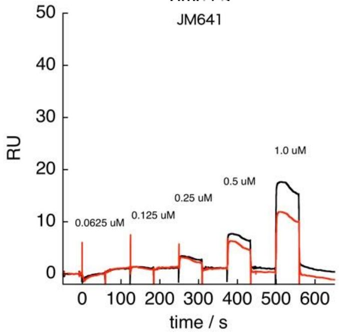

Figure S1. SPR single cycle kinetic analysis of the binding of **JM642** and derivatives to the r(CUG)$_9$ (black line) and r(CCG)$_9$ (red line). (a) **JM642** was added stepwise at concentrations of 6.3, 12.5, 25.0, 50.0, and 100 nM. (b~d) Compounds indicated in the left were added stepwise at concentrations of 0.063, 0.13, 0.25, 0.5, and 1.0 μM. (b) **JM676**, (c) **JM675**, (d) **JM665**, (e) **JM641**.
